# Assessing the Learning Curve for DMEK Using Post-Procedural Clinical Outcomes—Comparison of Four Different Surgeons during Two Different Periods

**DOI:** 10.3390/jcm12030811

**Published:** 2023-01-19

**Authors:** Emilia Sophie Stuhlmacher, Shady Suffo, Cristian Munteanu, Berthold Seitz, Loay Daas

**Affiliations:** Department of Ophthalmology, Saarland University Medical Center UKS, Kirrberger Straße 100, Building 22, 66421 Homburg/Saar, Germany

**Keywords:** DMEK, surgical learning curve, Fuchs’ endothelial corneal dystrophy, lamellar keratoplasty, corneal surgery

## Abstract

Purpose: Evaluating the learning curve of individual surgeons for Descemet Membrane Endothelial Keratoplasty (DMEK) and Triple-DMEK and assessing outcome with experience. Methods: The first 41 and the last 41 surgeries of each of the four surgeons were retrospectively included. Surgery duration and graft preparation time were recorded. Corrected distance visual acuity (CDVA, logMAR) and central corneal thickness (CCT, µm) were collected preoperatively after 6 and 12 months, as well as postoperative complications, e.g., re-bubbling or repeat penetrating keratoplasty. Results: Surgical duration for Triple-DMEK and DMEK decreased significantly by 21 min and 14 min between the two periods (*p* < 0.001; *p* < 0.001). Graft preparation time decreased significantly from 13.3 ± 5.2 min (95%CI 12.8–14.3) in period 1 to 10.7 ± 4.8 min (95%CI 10.2–11.4) in period 2 (*p* = 0.002). The postoperative changes in CDVA and CCT over both periods were not significant (*p* = 0.900; *p* = 0.263). The re-bubbling rate decreased significantly from 51.2% in period 1 to 26.2% in period 2 (*p* < 0.001). The repeat penetrating keratoplasty (PKP) was 7.3% in period 1 and 3.7% in period 2 (*p* = 0.146). Re-DMEK was necessary in 6.1% in period 1 and 4.9% in period 2 (*p* = 0.535). Several parameters showed significant differences between the surgeons in both periods (surgical duration: period 1: *p* < 0.001, period 2 *p* < 0.001; graft preparation: period 1: *p* < 0.001, period 2 *p* < 0.001). Conclusion: Significant decrease in surgery duration, graft preparation time, and the re-bubbling rate can be attributed to gained individual experience.

## 1. Introduction

Since Gerrit Melles first introduced the Descemet Membrane Endothelial Keratoplasty (DMEK) method in 2006 [1], it has become the gold standard in the surgical treatment of endothelial corneal dystrophies, especially Fuchs endothelial corneal dystrophy (FECD) in Germany [2]. At the Saarland University Medical Center, the method of DMEK was first introduced in 2013 [3]. Subsequently, a total of 1140 corneas have been transplanted using DMEK and Triple-DMEK from 2013 to 2021.

DMEK is a minimally invasive surgical method in which only the posterior part of the cornea, consisting of Descemet’s membrane (DM) and the endothelial layer, is transplanted [1]. In addition to DMEK, there is also a method called Triple-DMEK, where DMEK surgery is combined with simultaneous phacoemulsification with posterior chamber lens implantation. This combination is chosen especially for patients with cataract [4].

Advantages of DMEK over Descemet Stripping Automated Endothelial Keratoplasty (DSAEK) includes faster and better visual recovery, as well as higher patient satisfaction [5,6,7,8,9]. Furthermore, DMEK and Triple-DMEK demonstrate satisfactory results even in the long term [10,11]. As demonstrated in a study published in 2020, continuous improvement in visual acuity at 6 to 36 months postoperatively remained unchanged over a period of 5 years [10]. Additionally, the risk of rejection proved to be lower with DMEK than with DSAEK [12,13,14].

However, intra- and postoperative complications are not rare when DMEK is first implemented. The most common complication is postoperative graft detachment in 28.8% of cases (range: 2.4–82%) [6]. In the event of detachment, the anterior chamber is refilled with air or 20% SF6-gas (re-bubbling).

Dealing with the investigation of the learning curve in DMEK and Triple-DMEK, most previous published papers have discussed the postoperative patient results of a group of surgeons and did not compare individual surgeons. Previous studies demonstrated a steep learning curve [15,16,17], with reported difficulties including the preparation of the graft and unfolding of the graft in the recipient’s eye [18]. Over the years, various adaptions and standardizations regarding surgical technique have been established to improve outcomes and reduce complications [19,20]. Our findings add that the overall surgical duration and overall re-bubbling rate decreased significantly with increasing experience.

The aim of the present study was to retrospectively evaluate the learning curve of the individual surgeons for DMEK and Triple-DMEK at a University Medical Center and to determine whether a significant difference between the surgeons was evident. In addition, the purpose was to assess the evolution of the outcome with increasing experience.

## 2. Materials and Methods

In this retrospective study, the very first 41 and the last 41 surgeries of each of the four surgeons at the Department of Ophthalmology at the Saarland University Medical Center were compared. A total of 328 surgeries were included from July 2013 to December 2019, divided into two groups consisting of a total of 164 surgeries each. “Period 1” hereafter refers to the first set of 164 operations and “period 2” the last 164 operations. We intended to include the same number of surgeries for all 4 surgeons in both time periods. The chosen number of 41 surgeries in each period is due to the fact that the interval between the two periods was different for each surgeon and, accordingly, the surgeons with a shorter interval could perform only a smaller amount of primary versus final surgeries. Consequently, a smaller amount of data were available for analysis overall. Inclusion criteria included a diagnosis of FECD, pseudophakic bullous keratopathy, and graft failure after DSAEK. The study was conducted in accordance with the Declaration of Helsinki and was approved by the local ethical committee (Ethikkommision der Ärztekammer des Saarlandes) with the number “Bu 217/20”.

### 2.1. Main Outcome Measures 

Procedural parameters assessed were the graft preparation time and surgical duration, recorded from the surgical reports. 

The following patient outcomes were recorded preoperatively, 6 and 12 months postoperatively: corrected distance visual acuity (CDVA) as logMAR and central corneal thickness (CCT) measured with the Pentacam (Oculus GmbH, Wetzlar, Germany).

Postoperative complications were collected from the patient records including graft detachment, occurrence of cystoid macular edema (CME), re-bubbling rate, and repeat keratoplasty rates (DMEK, penetrating keratoplasty (PKP)). All parameters were entered into a Microsoft Access database (software version 2013).

### 2.2. Surgical Technique

The surgical procedure was performed according to the standard of the Saarland University Medical Center. Medication was administered according to the standardized DMEK regimen of the Department of Ophthalmology [21].

### 2.3. Statistical Analysis

SPSS software version 22 (IBM SPSS Statistics, International Business Machines Corporation (IBM), Armonk, NY, USA) was used to analyze the data. All values were expressed as mean ± standard deviation (SD), median (MED), and 95% confidence interval (95% CI).

The chi-square test was used to analyze the nominal scaled data. Multifactorial analysis of variance (three-way ANOVA) was used to compare the different parameters between surgeons and to compare the periods with each other. The significance level of all tests was set at 5%.

## 3. Results

In total, the study sample comprised 328 surgeries, 47.9% of them in male and 52.1% in female patients. Out of the total 328 surgeries, 199 were DMEK and 129 were Triple-DMEK (Table 1).

The diagnosis of FECD accounted for 98% of surgical indication. The average age of the patients was 70.0 ± 9.5 years, with a minimum of 32 years and a maximum of 95 years.

The time interval between the end of the first period and the beginning of the second period was 52 months for surgeon 1, since he did most of surgeries on his own after the introduction in this center, and only 8 months for surgeon 2. For surgeons 3 and 4, the intervals were 15 and 9 months, respectively. In the time between period 1 and period 2, surgeon 1 performed a total of 374 surgeries, of which 193 operations were DMEK and 181 were Triple-DMEK. Surgeon 2 performed 63 surgeries in the interim period, including 33 DMEK and 30 Triple-DMEK. For surgeon 3, 66 operations were counted between period 1 and 2, comprising 41 DMEK and 25 Triple-DMEK. Surgeon 4 performed 133 surgeries between period 1 and 2 (96 DMEK and 37 Triple-DMEK).

Surgeons 1 and 2 used air to fill the anterior chamber in all cases in period 1. In contrast, surgeon 3 filled the anterior chamber with air in period 1 in five cases and then switched to 20% SF6 gas. Surgeon 4 used 20% SF6 gas in his procedures from the beginning. In period 2, surgeon 2 remained the only surgeon using air, whereas the other surgeons used 20% SF6 gas.

### 3.1. Surgical Duration

**Figure 1:**
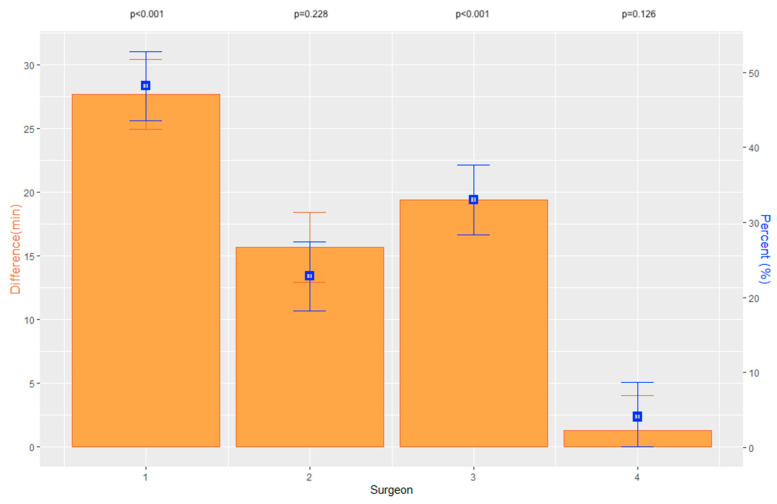
Graphs displaying the absolute difference of surgical duration of Triple-DMEK between period 1 and period 2 in minutes, including the standard error and *p*-values as well as the percentage difference for each surgeon.

**Figure 2:**
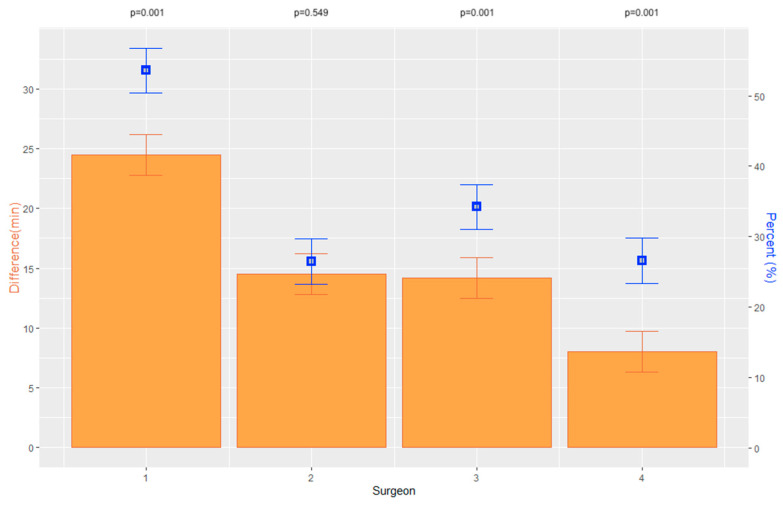
Graphs displaying absolute difference of surgical duration of DMEK of each surgeon between period 1 and 2 in minutes, including the standard error and *p*-values as well as the percentage difference for each surgeon.

Overall, the average duration of surgery for Triple-DMEK decreased significantly by 21 min from 58.7 ± 22.3 min (MED 51 min; 95% CI [49.4, 59.2]) to 37.7 ± 14.8 min (MED 36 min; 95% CI [34.2, 42.5]) (*p <* 0.001) between periods 1 and 2. For DMEK, the average duration of surgery decreased by 14 min from 41.1 ± 29.2 min (MED 36 min; 95% CI [38.2, 47.9]) to 27.0 ± 13.5 min (MED 25 min; 95% CI [23.3, 32.2]) between the two periods (*p <* 0.001). In two of the four surgeons, the duration of surgery for Triple-DMEK decreased significantly between the two periods (Table 2; Figure 1). In DMEK, the duration of surgery decreased significantly between the two periods for three of the four surgeons (Table 2; Figure 2). Overall, a significant difference between surgeons was evident in period 1 (*p <* 0.001) and period 2 (*p <* 0.001) for Triple-DMEK as well as for DMEK (period 1: *p =* 0.002; period 2: *p <* 0.001). Surgeon 4 was significantly faster in Triple-DMEK than surgeons 1, 2 and 3 in period 1 (*p =* 0.003; *p <* 0.001; *p =* 0.013). In period 2, surgeons 1 and 4 demonstrated a significantly shorter surgical duration in Triple-DMEK and DMEK compared to surgeon 2 (Triple-DMEK: *p <* 0.001; *p <* 0.001; DMEK: *p <* 0.001; *p <* 0.001).

### 3.2. Graft Preparation Time

**Figure 3:**
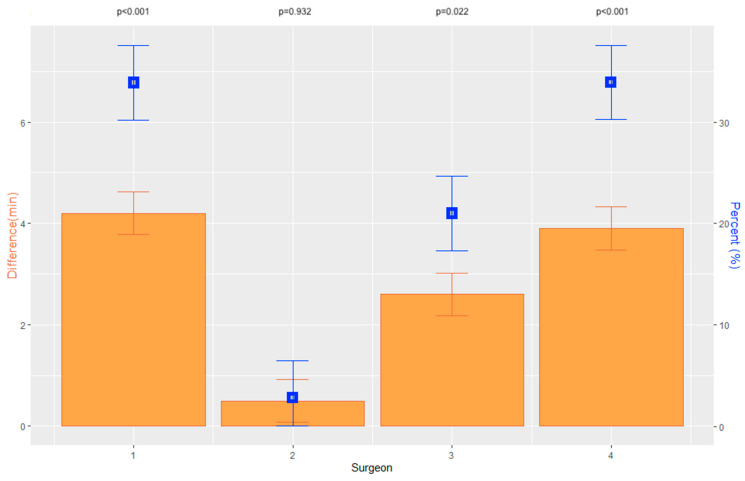
Graphs displaying the absolute difference of graft preparation time of each surgeon between periods 1 and 2 in minutes, including the standard error and *p*-values as well as the percentage difference for each surgeon.

Overall, the average graft preparation time decreased significantly from 13.3 ± 5.2 min (MED 13 min; 95% CI [12.8, 14.3]) period 1 to 10.7 ± 4.8 min (MED 9 min; 95% CI [10.2, 11.4]) in period 2 (*p* < 0.001) (Table 2; Figure 3).

Overall, a significant difference between the surgeons was observed in period 1 (*p* < 0.001) as well as in period 2 (*p* < 0.001). 

In both periods 1 and 2, surgeons 1, 3, and 4 were significantly quicker than surgeon 2 (Table 2). 

### 3.3. Visual Acuity

On average, preoperative CDVA was 0.51 ± 0.23 logMAR (MED 0.52 logMAR; 95% CI [0.46, 0.55] at period 1, improving significantly to 0.31 ± 0.33 logMAR (MED 0.22 logMAR; 95% CI [0.26, 0.35]) at 6 months postoperatively (*p* < 0.001), and further to 0.22 ± 0.24 logMAR (MED 0.22 logMAR; 95% CI [0.17, 0.27]) at 1 year (*p* = 0.042). 

During period 2, the mean preoperative CDVA was 0.50 ± 0.24 logMAR (MED 0.40 logMAR; 95% CI [0.46, 0.55]) decreasing significantly to 0.32 ± 0.24 logMAR (MED 0.22 logMAR; 95% CI [0.26, 0.35]) (*p* < 0.001) at 6 months and to 0.22 ± 0.39 logMAR (MED 0.22 logMAR; 95% CI [0.16, 0.27]) (*p* = 0.04) at 1 year.

No significant difference was found between the two periods when comparing the preoperative (*p* = 0.930), 6-month (*p* = 0.947), and 1-year results (*p* = 0.900).

Significant differences of CDVA could not be detected between the surgeons in both periods at any examination (Table 3).

### 3.4. Central Corneal Thickness

Period 2 = the last 41 DMEK procedures of the respective surgeon in his/her career.

Overall, the average CCT was 656 ± 89 µm (MED 644 µm; 95% CI [642, 670]) preoperatively, decreasing significantly by 16.3% to 549 ± 77 µm (MED 538 µm) (*p* < 0.001) 6 months postoperatively. One year postoperatively, CCT was measured as being 15.4% (555 ± 100 µm; MED 536 µm) lower than the preoperative CCT (*p* = < 0.001), without significant improvement between 6 months and one year. In period 2, the preoperative CCT was 636 ± 102 µm (MED 615 µm; 95% CI [624, 648]), improving significantly by 14.6% to 543 ± 65 µm (MED 533 µm; 95% CI [530, 555]) at 6 months (*p* < 0.001), and remaining stable without significant improvement at one year, measuring 541 ± 56 µm (14.9%) (MED 529 µm; 95% CI [529, 556]) (*p* = 1.0).

No significant difference between the two periods was observed at any time point (preOP: *p* = 0.059; 6 months: *p* = 0.453; 1 year: *p* = 0.263).

Significant differences were found between the surgeons at the one year control in both periods (Table 4).

### 3.5. Complications

Overall, the re-bubbling rate decreased significantly from 51.2% in period 1 to 26.2% in period 2 (p < 0.001). In period 1, the re-bubbling rate of surgeons 1 and 2 was higher than surgeons 3 and 4 (*p* = 0.006), while there was no difference between the surgeons in period 2 (*p* = 0.412).

In period 1, postoperative repeat PKP was performed in 12 of 164 cases. In period 2, 6 of 164 cases required a repeat PKP. There was no significant difference between the two periods overall (*p* = 0.146). Surgeon 4 significantly reduced the rate of repeat PKP between period 1 and period 2 (*p* = 0.021).

A similar rate of repeat DMEK was performed in 10 of 164 cases in period 1 and in 8 of 164 cases in period 2 (*p* = 0.535). There was also no significant difference between the two periods for the individual surgeons (Table 5).

Postoperatively, cystoid macular edema developed in 4 of 164 cases in period 1 and in 7 of 164 cases in period 2 (*p* = 0.358), without significant difference between the surgeons (period 1: *p* = 0.105; period 2: *p* = 0.258).

## 4. Discussion

The aim of this study was to survey the learning curve of surgeons using the aforementioned parameters and to analyze the evolution of the outcomes. The present study compared the outcomes of four individual surgeons for their first 41 and their last 41 surgeries (2013–2019). The surgical duration as well as the graft preparation time were also included in the evaluation, which has only been considered in a few studies so far [15,22].

Introducing the DMEK and Triple-DMEK techniques poses a challenge to surgeons. It should be noted that surgeon 1 performed their first DMEK at Saarland University Medical Center and, therefore, had no experience of colleagues to draw on, facing the challenges of graft preparation and graft unfolding on his own. The other surgeons at the University Medical Center were initially able to learn their surgical skills in a wetlab [23] and subsequently benefited from the experience gained by the first surgeon, who trained all following DMEK surgeons individually. This information is of importance when comparing the learning curve of the individual surgeons.

Initially, surgeon 1 required 57.5 ± 17.6 min per Triple-DMEK procedure and 45.7 ± 30.1 min per DMEK, whereas, in period 2, this surgeon needed only 29.8 ± 11.9 min for Triple-DMEK and 21.2 ± 9.9 min for DMEK. In addition, surgeon 4 required only 30.1 min ± 11.9 min for DMEK from the beginning, improving significantly to 22.1 ± 8.7 min. Wubbels et al. [22] found that the surgery duration averaged 88 ± 16 min at the Rotterdam Eye Hospital without improvement with increasing experience. However, it must be taken into account that Wubbels et al.’s study included only a total of 40 surgeries performed by two surgeons. Most of the surgeons in the present study showed a significant improvement in Triple-DMEK and DMEK between the two periods, which suggests that a learning curve could be derived from the total surgery duration.

The graft preparation poses difficulties for the surgeons, especially at the beginning. Possible sources of error include tearing of the Descemet’s membrane and incorrect or even absent markings for orientation, which can lead to increased complications during the operation and also postoperatively [21,24,25]. The danger of upside-down localization of the graft, which can occur predominantly in older donor corneas, is particularly important here [26].

On average, the preparation time was 13.3 ± 5.1 min in period 1 and 10.7 ± 4.7 min in period 2. The change over the two periods was significant. It was striking that the period did not change significantly for surgeon 2 and that he constantly performed slower compared to the other surgeons. 

However, the results of the three other surgeons demonstrate a learning curve, with improved graft preparation time. This aligns with the findings of Debellmanière et al. [15], who showed an average preparation time of 20.2 ± 10.6 min and a reduction to 6.2 ± 5.2 min for their last 27 cases [15].

The collected data of visual acuity after 6 months were 0.31 ± 0.32 logMAR for period 1 and 0.31 ± 0.24 logMAR in period 2 overall, without a significant difference between the two periods (*p* = 0.930). Visual acuity improved to 0.22 ± 0.24 logMAR at 1 year postoperatively in period 1 and to 0.22 ± 0.39 logMAR at 1 year in period 2, again without any difference between the periods (*p* = 0.947). The study by Dunker et al. [27] showed a visual acuity of 0.15 logMAR at 6 months and 0.12 logMAR at one year postoperatively. In a study by Birbal et al. [10], the mean CDVA was 0.11 ± 0.27 logMAR at 6 months and 0.06 ± 0.15 logMAR at 12 months. It should be noted that, unlike in these two studies, our study did not exclude patients with other ocular comorbidities limiting visual acuity such as macular degeneration or glaucoma, and our results for visual acuity are therefore inferior to other published studies. As acknowledged by other studies [28,29,30], however, an evaluation of visual acuity alone does not permit conclusions regarding the learning curve of the surgeons to be drawn.

CCT decreased on average by 15.4% from 656 ± 89 μm to 555 ± 100 μm during the first year in period 1. In period 2, CCT reduced from 636 μm to 541 μm, resulting in a rate of 14.9%. Considering Birbal’s study, the results of the present study are consistent. The CCT decreased by 20% within one year, from 667 ± 92 μm preoperatively to 527 ± 40 μm [10]. The study of Schrittenlocher also showed similar values, with the CCT reducing from 711 ± 70 μm to 570 ± 76 μm [30].

The reduction in CCT is dependent on the physiological pumping function of the endothelial cells, which reduces stromal oedema and decreases the thickness of the cornea. In our findings, all surgeons showed good postoperative results in both periods, and, therefore, CCT, again, is not a good parameter to assess the learning curve of individual surgeons.

Our findings regarding the re-bubbling rates confirm previous publications’ findings as well, though rates vary greatly between studies [15,27,28,29]. Unlike other studies, we included the very first attempts of surgeon 1 before DMEK and Triple-DMEK became well-established protocols at our center. Rodríguez et al. [31] found a re-bubbling rate of 3%; however, the first 25 operations were excluded. Furthermore, the rate of re-bubbling depends on the individual surgeon and their experience, which includes developing better judgment regarding the necessity of re-entering air or gas into the anterior chamber to prevent future graft detachment. In our department, we place emphasis on a high rate of complete graft attachment, which results in a comparatively high re-bubbling rate, especially during period 1.

The work by Dapena et al. demonstrated a correlation between experience and a reduction in the rate of re-bubbling. The occurrence of re-bubbling improved from 11.1% in the first cases to 2.2% during the last 44 cases [28].

In a review, the average rate was reported to be 28.8% (range: 2.4–82%) and also proved that a reduction in the re-bubbling rate is associated with increasing experience [6]. The present results of the re-bubbling rate in period 2 are comparable within the range of re-bubbling rates found in other studies, indicating that the re-bubbling rate clearly depends on the surgeons’ experience.

Another factor in the improved re-bubbling rate is that the first DMEK operations of surgeons 1, 2, and 3 at our department used air to fill the anterior chamber, which is known to cause more frequent re-bubbling. Only at a later stage were adjustments made by using 20% SF_6_ gas [20].

In the present study, the Triple-DMEK during the first period of surgeons 1 and 2 were performed with air inflation, while surgeon 4 used 20% SF_6_ gas from the beginning. Therefore, one could possibly conclude that the re-bubbling rate of 34.1% for surgeon 4 was lower from the beginning because he already benefited from the modified surgical technique. Schrittenlocher et al. reported that, once air was entered, the re-bubbling rate was 68% and decreased to 16% when SF_6_ gas was introduced [30].

In the present study, the rate of repeat keratoplasties was kept very low from the beginning. This was also observed in a study by Pereira et al. [32], wherein only 5.7% of the first cases required repeat keratoplasties. Pereira et al. explained the low number with the fact that the surgeons were supported by an experienced surgeon during the first operations. This reason could also be a possible explanation for the low rate in both periods in our case. In another paper by Dapena et al. [28], they observed that the need for repeat keratoplasty decreased with increasing experience. Initially, the rate was 22.2% in the first 45 cases and decreased to 2.2% after the first 90 cases [28]. 

Although the overall difference in repeat keratoplasties between the two periods was not significant, in the present study, it can be assumed that, with increasing experience, the frequency of repeat keratoplasties decreases.

The incidence of postoperative cystoid macular edema reported in the literature is 10% [19]. This complication can be significantly reduced by administration of topical steroids after the procedure [33]. Postoperative drug treatment with steroids is also standard procedure at Saarland University Medical Center [21]. In this study, the occurrence of cystoid macular edema was 2.4% in period 1 and 4.3% in period 2. This rate is lower compared to the results of other authors: Kocaba showed cystoid macular oedema in 13.6% of cases and, in Heinzelmann’s study, the rate was 13% [33,34]. Based on our results, no statement can be made about a potential learning curve in regard to the occurrence of cystoid macular edema.

In summary, all four DMEK surgeons underwent a learning curve, demonstrated by the surgical duration, the graft preparation time, and the re-bubbling rate. With increasing experience, the graft preparation time as well as the surgical duration decreased. Additionally, the surgeons became more experienced in recognizing the need for reintroducing air/gas into the anterior chamber, lowering the re-bubbling rate. An interesting question for future studies would be whether the learning curves differ between DMEK and Triple-DMEK.

In conclusion, the gained knowledge about the learning curve should encourage surgeons to learn and apply the technique of DMEK and Triple-DMEK despite possible initial complications. Surgeons can achieve very good results early on if they practice repeatedly in a designated DMEK wetlab and are mentored by an experienced surgeon who communicates their initial complications and pitfalls well to less experienced colleagues.

## Figures and Tables

**Table 1:** Individual numbers of surgeries for 4 surgeons in period 1 and period 2 [Period 1 = first 41 surgeries of each surgeon; Period 2 = last 41 surgeries of each surgeon].

		Surgeon				Total
		1	2	3	4	
Period 1	Triple-DMEK	18	23	14	9	64
	DMEK	23	18	27	32	100
	Total	41	41	41	41	164
Period 2	Triple-DMEK	20	17	16	12	65
	DMEK	21	24	25	29	99
	Total	41	41	41	41	164

**Table 2:** Surgical data.

			Surgeon 1	Surgeon 2	Surgeon 3	Surgeon 4	*p*-Value
Surgical duration, min	Triple-DMEK	Period 1	57.5 ± 17.6 (52)	68.8 ± 25.6 (58)	58.8 ± 16.8 (57.5)	32.2 ± 5 (32)	<0.001
		Period 2	29.8 ± 11.9 (28)	53.1 ± 12.0 (50)	39.4 ± 8.4 (37)	30.9 ± 16 (29)	<0.001
		*p*-value	<0.001	0.228	<0.001	0.126	
	DMEK	Period 1	45.7 ± 30.1 (40)	54.8 ± 49.6 (40.5)	41.5 ± 16.8 (37)	30.1 ± 11.9 (25)	0.002
		Period 2	21.2 ± 9.8 (18)	40.3 ± 17.5 (37)	27.3 ± 9.5 (24)	22.1 ± 8.7 (20)	<0.001
		*p*-value	0.001	0.549	0.001	0.001	
Graft preparation time, min		Period 1	12.4 ± 4.1 (13)	17.9 ± 4.0 (16)	12.4 ± 5.4 (12)	11.5 ± 4.3 (11)	<0.001
		Period 2	8.2 ± 2.1 (8)	17.4 ± 4.3 (17)	9.8 ± 2.4 (9)	7.6 ± 2.2 (8)	<0.001
		*p*-value	<0.001	0.932	0.022	<0.001	

All values are mean values ± standard deviation (median); Period 1 = the 41 first DMEK procedures of the respective surgeon in his/her career; Period 2 = the last 41 DMEK procedures of the respective surgeon in his/her career.

**Table 3:** Clinical results * after DMEK and Triple-DMEK for each surgeon—CDVA (logMAR).

		Surgeon 1	Surgeon 2	Surgeon 3	Surgeon 4	*p*-Value
Period 1	preOP	0.6 ± 0.3 (0.5)	0.5 ± 0.2 (0.5)	0.5 ± 0.3 (0.4)	0.5 ± 0.2 (0.4)	0.345
	6 Months	0.3 ± 0.4 (0.2)	0.3 ± 0.3 (0.2)	0.3 ± 0.3 (0.2)	0.3 ± 0.3 (0.2)	0.851
	1 Year	0.2 ± 0.2 (0.1)	0.3 ± 0.2 (0.2)	0.2 ± 0.3 (0.2)	0.3 ± 0.3 (0.2)	0.275
Period 2	preOP	0.5 ± 0.2 (0.4)	0.6 ± 0.3 (0.5)	0.5 ± 0.3 (0.4)	0.5 ± 0.2 (0.5)	0.208
	6 Months	0.3 ± 0.2 (0.2)	0.4 ± 0.3 (0.3)	0.3 ± 0.2 (0.2)	0.3 ± 0.2 (0.3)	0.553
	1 Year	0.2 ± 0.3 (0.2)	0.2 ± 0.7 (0.2)	0.2 ± 0.2 (0.2)	0.2 ± 0.4 (0.2)	0.844

* CDVA, corrected distance visual acuity; all values are mean values ± standard deviation (median), Period 1 = the 41 first DMEK procedures of the respective surgeon in his/her career; Period 2 = the last 41 DMEK procedures of the respective surgeon in his/her career.

**Table 4:** Clinical results * after DMEK and Triple-DMEK for each surgeon—CCT (µm).

		Surgeon 1	Surgeon 2	Surgeon 3	Surgeon 4	*p*-Value
Period 1	preOP	660 ± 87 (645)	656 ± 67 (656)	666 ± 108 (639)	642 ± 94 (633)	0.681
	6 Months	550 ± 68 (542)	557 ± 89 (549)	529 ± 71 (522)	560 ± 76 (545)	0.295
	1 Year	528 ± 42 (529)	605 ± 164 (548)	530 ± 45 (534)	551 ± 74 (527)	0.01
Period 2	preOP	596 ± 50 (595)	675 ± 129 (639)	658 ± 71 (637)	616 ± 49 (615)	<0.001
	6 Months	532 ± 57 (529)	530 ± 55 (526)	562 ± 81 (542)	547 ± 61 (540)	0.134
	1 Year	518 ± 41 (513)	572 ± 79 (562)	548 ± 49 (536)	532 ± 38 (528)	<0.001

* CCT, central corneal thickness; all values are mean values ± standard deviation (median); Period 1 = the 41 first DMEK procedures of the respective surgeon in his/her career.

**Table 5:** Complications after DMEK for each surgeon.

		Surgeon 1	Surgeon 2	Surgeon 3	Surgeon 4
Re-bubbling	Period 1	56.1%	70.7%	43.9%	34.1%
	Period 2	29.3%	34.1%	22%	19.5%
	*p*-value	0.014	0.001	0.034	0.135
Re-PKP	Period 1	3	3	1	5
	Period 2	1	3	2	0
	*p*-value	0.305	1.000	0.556	0.021
Re-DMEK	Period 1	2	4	1	3
	Period 2	1	4	2	1
	*p*-value	0.556	1.000	0.556	0.347
CME *	Period 1	0	3	0	1
	Period 2	0	3	3	1
	*p*-value		1.000	0.078	1.000

* CME = cystoid macular edema; n or %; Period 1 = the 41 first DMEK procedures of the respective surgeon in his/her career; Period 2 = the last 41 DMEK procedures of the respective surgeon in his/her career.

## Data Availability

The data presented in this study are available on request from the corresponding author. The data are not publicly available due to restrictions containing information that could compromise the privacy of research participants.

## References

[1] Melles G.R.J., Ong T.S., Ververs B., van der Wees J. (2006). Descemet Membrane Endothelial Keratoplasty (DMEK). Cornea.

[2] Flockerzi E., Maier P., Böhringer D., Reinshagen H., Kruse F., Cursiefen C., Reinhard T., Geerling G., Torun N., Seitz B. (2018). Trends in Corneal Transplantation from 2001 to 2016 in Germany: A Report of the DOG-Section Cornea and Its Keratoplasty Registry. Am. J. Ophthalmol..

[3] Lang S.J., Bischoff M., Böhringer D., Seitz B., Reinhard T. (2014). Analysis of the Changes in Keratoplasty Indications and Preferred Techniques. PLoS ONE.

[4] Girbardt C., Wiedemann P., Nestler A. (2016). Triple-Descemet-Membran-Endothel-Keratoplastik [Triple Descemet Membrane Endothelial Keratoplasty. Indications, Variations and Results]. Ophthalmologe.

[5] Chamberlain W., Lin C.C., Austin A., Schubach N., Clover J., McLeod S.D., Porco T.C., Lietman T.M., Rose-Nussbaumer J. (2019). Descemet Endothelial Thickness Comparison Trial: A Randomized Trial Comparing Ultrathin Descemet Stripping Automated Endothelial Keratoplasty with Descemet Membrane Endothelial Keratoplasty. Ophthalmology.

[6] Deng S.X., Lee W.B., Hammersmith K.M., Kuo A.N., Li J.Y., Shen J.F., Weikert M.P., Shtein R.M. (2018). Descemet Membrane Endothelial Keratoplasty: Safety and Outcomes: A Report by the American Academy of Ophthalmology. Ophthalmology.

[7] Goldich Y., Showail M., Avni-Zauberman N., Perez M., Ulate R., Elbaz U., Rootman D.S. (2015). Contralateral Eye Comparison of Descemet Membrane Endothelial Keratoplasty and Descemet Stripping Automated Endothelial Keratoplasty. Am. J. Ophthalmol..

[8] Maier A.-K.B., Gundlach E., Gonnermann J., Klamann M.K.J., Bertelmann E., Rieck P.W., Joussen A.M., Torun N. (2015). Retrospective Contralateral Study Comparing Descemet Membrane Endothelial Keratoplasty with Descemet Stripping Automated Endothelial Keratoplasty. Eye.

[9] Phillips P.M., Phillips L.J., Muthappan V., Maloney C.M., Carver C.N. (2017). Experienced DSAEK Surgeon’s Transition to DMEK: Outcomes Comparing the Last 100 DSAEK Surgeries with the First 100 DMEK Surgeries Exclusively Using Previously Published Techniques. Cornea.

[10] Birbal R.S., Ni Dhubhghaill S., Bourgonje V.J.A., Hanko J., Ham L., Jager M.J., Böhringer S., Oellerich S., Melles G.R.J. (2020). Five-Year Graft Survival and Clinical Outcomes of 500 Consecutive Cases After Descemet Membrane Endothelial Keratoplasty. Cornea.

[11] Schlögl A., Tourtas T., Kruse F.E., Weller J.M. (2016). Long-Term Clinical Outcome after Descemet Membrane Endothelial Keratoplasty. Am. J. Ophthalmol..

[12] Anshu A., Price M.O., Price F.W. (2012). Risk of Corneal Transplant Rejection Significantly Reduced with Descemet’s Membrane Endothelial Keratoplasty. Ophthalmology.

[13] Hos D., Tuac O., Schaub F., Stanzel T.P., Schrittenlocher S., Hellmich M., Bachmann B.O., Cursiefen C. (2017). Incidence and Clinical Course of Immune Reactions after Descemet Membrane Endothelial Keratoplasty: Retrospective Analysis of 1000 Consecutive Eyes. Ophthalmology.

[14] Steven P., Hos D., Heindl L.M., Bock F., Cursiefen C. (2013). Immunreaktionen nach DMEK, DSAEK und DALK [Immune reactions after DMEK, DSAEK and DALK]. Klin. Mon. Für Augenheilkd..

[15] Debellemanière G., Guilbert E., Courtin R., Panthier C., Sabatier P., Gatinel D., Saad A. (2017). Impact of Surgical Learning Curve in Descemet Membrane Endothelial Keratoplasty on Visual Acuity Gain. Cornea.

[16] Koo E.H., Pineda R., Afshari N., Eghrari A. (2020). Learning Descemet Membrane Endothelial Keratoplasty: A Survey of U.S. Corneal Surgeons. Cornea.

[17] Borroni D., Rocha de Lossada C., Parekh M., Gadhvi K., Bonzano C., Romano V., Levis H.J., Tzamalis A., Steger B., Rechichi M. (2021). Tips, Tricks, and Guides in Descemet Membrane Endothelial Keratoplasty Learning Curve. J. Ophthalmol..

[18] Zafar S., Parker J.S., de Kort C., Melles G., Sikder S. (2019). Perceived Difficulties and Barriers to Uptake of Descemet’s Membrane Endothelial Keratoplasty among Surgeons. Clin. Ophthalmol..

[19] Bachmann B., Schrittenlocher S., Schaub F., Siebelmann S., Matthaei M., Cursiefen C. (2017). DMEK: Probleme Vermeiden, Erkennen, Lösen [Complications of DMEKeratoplasty: Avoid, Recognize and Treat]. Klin. Mon. Für Augenheilkd..

[20] Terry M.A., Straiko M.D., Veldman P.B., Talajic J.C., VanZyl C., Sales C.S., Mayko Z.M. (2015). Standardized DMEK Technique: Reducing Complications Using Prestripped Tissue, Novel Glass Injector, and Sulfur Hexafluoride (SF6) Gas. Cornea.

[21] Seitz B., Daas L., Flockerzi E., Suffo S. (2020). “Descemet membrane endothelial keratoplasty” DMEK-Spender und Empfänger Schritt für Schritt [Descemet membrane endothelial keratoplasty DMEK—Donor and recipient step by step]. Ophthalmologe.

[22] Wubbels R.J., Remeijer L., Engel A., van Rooij J. (2020). The Learning Curve for Descemet Membrane Endothelial Keratoplasty Performed by Two Experienced Corneal Surgeons: A Consecutive Series of 40 Cases. Acta Ophthalmol..

[23] Seitz B., Daas L., Bischoff-Jung M., Szentmáry N., Suffo S., El-Husseiny M., Viestenz A., Milioti G. (2018). Anatomy-Based DMEK Wetlab in Homburg/Saar: Novel Aspects of Donor Preparation and Host Maneuvers to Teach Descemet Membrane Endothelial Keratoplasty. Clin. Anat..

[24] Birbal R.S., Baydoun L., Ham L., Miron A., van Dijk K., Dapena I., Jager M.J., Böhringer S., Oellerich S., Melles G.R.J. (2020). Effect of Surgical Indication and Preoperative Lens Status on Descemet Membrane Endothelial Keratoplasty Outcomes. Am. J. Ophthalmol..

[25] Kruse F.E., Laaser K., Cursiefen C., Heindl L.M., Schlötzer-Schrehardt U., Riss S., Bachmann B.O. (2011). A Stepwise Approach to Donor Preparation and Insertion Increases Safety and Outcome of Descemet Membrane Endothelial Keratoplasty. Cornea.

[26] Bachmann B.O., Laaser K., Cursiefen C., Kruse F.E. (2010). A Method to Confirm Correct Orientation of Descemet Membrane during Descemet Membrane Endothelial Keratoplasty. Am. J. Ophthalmol..

[27] Dunker S.L., Veldman M.H.J., Winkens B., van den Biggelaar F.J.H.M., Nuijts R.M.M.A., Kruit P.J., Dickman M.M. (2020). Dutch Cornea Consortium Real-World Outcomes of DMEK: A Prospective Dutch Registry Study. Am. J. Ophthalmol..

[28] Dapena I., Ham L., Droutsas K., van Dijk K., Moutsouris K., Melles G.R.J. (2011). Learning Curve in Descemet’s Membrane Endothelial Keratoplasty: First Series of 135 Consecutive Cases. Ophthalmology.

[29] Oellerich S., Baydoun L., Peraza-Nieves J., Ilyas A., Frank L., Binder P.S., Melles G.R.J. (2017). Multicenter Study of 6-Month Clinical Outcomes after Descemet Membrane Endothelial Keratoplasty. Cornea.

[30] Schrittenlocher S., Schaub F., Hos D., Siebelmann S., Cursiefen C., Bachmann B. (2018). Evolution of Consecutive Descemet Membrane Endothelial Keratoplasty Outcomes Throughout a 5-Year Period Performed by Two Experienced Surgeons. Am. J. Ophthalmol..

[31] Rodríguez-Calvo-de-Mora M., Quilendrino R., Ham L., Liarakos V.S., van Dijk K., Baydoun L., Dapena I., Oellerich S., Melles G.R.J. (2015). Clinical Outcome of 500 Consecutive Cases Undergoing Descemet’s Membrane Endothelial Keratoplasty. Ophthalmology.

[32] Pereira N.C., Gomes J.Á.P., Moriyama A.S., Chaves L.F., Forseto A.D.S. (2019). Descemet Membrane Endothelial Keratoplasty Outcomes During the Initial Learning Curve of Cornea Fellows. Cornea.

[33] Heinzelmann S., Maier P., Böhringer D., Hüther S., Eberwein P., Reinhard T. (2015). Cystoid Macular Oedema Following Descemet Membrane Endothelial Keratoplasty. Br. J. Ophthalmol..

[34] Kocaba V., Mouchel R., Fleury J., Marty A.-S., Janin-Manificat H., Maucort-Boulch D., Burillon C. (2018). Incidence of Cystoid Macular Edema After Descemet Membrane Endothelial Keratoplasty. Cornea.

